# Courtship and distress ultrasonic vocalizations are disrupted in a mouse model of Angelman syndrome

**DOI:** 10.21203/rs.3.rs-5953744/v1

**Published:** 2025-02-11

**Authors:** Caleigh D. Guoynes, Grace Pavalko, Michael S. Sidorov

**Affiliations:** Children’s National Hospital; Children’s National Hospital; Children’s National Hospital

**Keywords:** Angelman syndrome, Ube3a, ultrasonic vocalizations, behavior

## Abstract

**Background:**

Angelman syndrome (AS) is a single-gene neurodevelopmental disorder caused by loss of function of the maternal copy of the *UBE3A* gene. Nearly all individuals with AS lack speech, resulting in major impacts on daily life for patients and caregivers. To evaluate new therapies for AS, it is crucial to have a mouse model that characterizes meaningful clinical features. Vocalizations are used in many contexts in mice, including pup retrieval, social interactions, courtship, and distress. Previous work in the *Ube3a*^*m*−*/p*+^ mouse model of AS found abnormalities in the number of ultrasonic vocalizations (USVs) mice produced during pup isolation and same-sex social interactions. Here, we evaluated *Ube3a*^*m*−*/p*+^ vocalizations during courtship and distress. Quantifying USVs in these contexts enables comparison of USVs in social (courtship) and non-social (distress) settings. In addition, we assessed the utility of incorporating USV testing into existing *Ube3a*^*m*−*/p*+^ mouse behavioral assessments used to evaluate potential AS treatments.

**Methods:**

We used a three-chamber social preference test for courtship vocalizations and a tail suspension test for distress vocalizations in adult wild-type (WT) and *Ube3a*^*m*−*/p*+^ littermates, and quantified USV properties using the program DeepSqueak. Next, mice performed an established *Ube3a*^*m*−*/p*+^ behavioral battery that included rotarod, open field, marble burying, and nest building. We used principal component analysis to evaluate the value of USV testing in the context of other behaviors.

**Results:**

In both social courtship and nonsocial distress behavioral paradigms, *Ube3a*^*m*−*/p*+^ mice made fewer USVs compared to WT mice. Spectral properties of USVs were abnormal in *Ube3a*^*m*−*/p*+^ mice on the courtship test but mostly typical on the distress test. Including USVs in the *Ube3a*^*m*−*/p*+^ mouse behavior battery increased the distance between *Ube3a*^*m*−*/p*+^ and WT clusters in principal component space.

**Conclusions:**

*Ube3a* ^*m*−*/p*+^ mice have difficulty producing USVs in social and nonsocial contexts. Spectral properties of USVs are most impacted in the social courtship context. Adding USVs to the *Ube3a*^*m*−*/p*+^ behavior battery may improve sensitivity to detect group differences and changes in communication.

## BACKGROUND

Angelman syndrome (AS) is a neurodevelopmental disorder characterized by lack of speech, motor and cognitive impairment, seizures, altered sleep, and differences in social behavior (1, 2). AS occurs when the maternal copy of the *Ube3a* gene is not functional in neurons due to deletion, mutation, or imprinting defect on the maternal chromosome or paternal uniparental disomy (3–5). In all genetic subtypes of AS, the paternal copy of *UBE3A* in neurons is intact but epigenetically silenced by a long non-coding antisense RNA (*UBE3A-ATS*) (6–9). Drug therapies designed to unsilence the paternal copy of *Ube3a* have been extraordinarily successful in mice (10–20) and a subset of these compounds are now in clinical trials (21). Beyond targeting paternal *UBE3A*, several other mechanism-based approaches, including AAV-based gene transfer and downstream approaches, are in various stages of development (22–26). Developing strong animal models of meaningful AS phenotypes will provide value for preclinical drug testing, regardless of the mechanism of potential treatment.

Impaired communication is the most difficult and pervasive AS symptom throughout childhood, negatively impacting quality of life and independence (27). Expressive communication is more severely affected than receptive communication: nearly all children with AS lack speech entirely, and as a result, many rely on single syllable vowel sounds and gestures or symbols to communicate (28–34). Impaired speech in AS is likely not due to a lack of social interest or desire to communicate, as children with AS have strong social skills in other nonverbal domains (e.g. good eye contact with caregivers, ability to initiate social contact with familiar adults, can readily distinguish between positive and neutral faces in unfamiliar adults, and smile and laugh very frequently during social interactions) (28–30, 35–41). Additionally, intellectual disability alone likely does not account for the lack of speech in children with AS; rather, it is thought to be driven by motor dysfunction characteristic of AS that also manifests in speech production (30, 31, 42–44).

Modeling impaired communication in preclinical rodent models of AS is inherently challenging but also critically important when evaluating therapies. *UBE3A* imprinting is conserved in mice (45), and the well-established *Ube3a*^*m*−*/p*+^ mouse model (46) is widely used to assess behavioral response to new treatments. Often, treatments are evaluated preclinically in *Ube3a*^*m*−*/p*+^ mice using a standardized behavioral testing battery that includes weight, rotarod, open field, marble burying, and nest building tests (12, 15, 17, 47–52). The advantage of this battery (53) is that it is remarkably reliable and repeatable across cohorts of mice and different labs; however, a limitation of this battery is that it does not assess communication. Here, we test the hypotheses that vocalizations are abnormal in *Ube3a*^*m*−*/p*+^ mice, and that vocalization test(s) can be incorporated into the existing *Ube3a*^*m*−*/p*+^ mouse behavioral battery to enhance its value.

Mice produce ultrasonic vocalizations (USVs) in a wide range of behavioral contexts that can be quantified in mouse models of neurodevelopmental disorders. Mouse USVs are not speech, but USVs are used to communicate during social interactions between pups and parents (54–57), novel conspecifics (58), and potential mates (59). Mice can also vocalize during nonsocial interactions, such as periods of distress (60–63). USVs have been widely used to characterize vocal production and communication deficits in many mouse models of neurodevelopmental disorders, including Down syndrome, Fragile X syndrome, and Prader-Willi syndrome (64–70). For AS, *Ube3a*^*m*−*/p*+^ mice show abnormal USVs in some social contexts, including abnormal development of *Ube3a*^*m*−*/p*+^ pup calls during a maternal isolation test (71, 72) and fewer USVs during adult female-female social interactions (42). Here, we sought to extend our understanding of *Ube3a*^*m*−*/p*+^ mouse USVs to two additional behavioral environments where prior work has been limited: courtship and distress. The strength of the courtship assay is that it reliably induces complex vocalizations that function to impress and attract a female as a mate (59, 73, 74). These complex vocalizations are harder to produce than other types of social calls and may provide additional insight into how social vocalizations are disrupted in *Ube3a*^*m*−*/p*+^ mice. Strengths of the distress test are that it evaluates nonsocial vocal production (75, 76) and is a fast, simple test that could be easily added to the existing *Ube3a*^*m*−*/p*+^ behavioral battery. Together, evaluating vocalizations in social courtship and nonsocial distress paradigms will expand our understanding of how the communication domain is impacted in *Ube3a*^*m*−*/p*+^ behavior.

Our prior work used multidimensional analysis to summarize mouse behavioral performance on the *Ube3a*^*m*−*/p*+^ behavior battery as a single severity score (77). This approach uses principal component analysis (PCA) to reduce the dimensionality of a behavioral dataset and *k*-means clustering to group animals by behavioral performance. We found that multidimensional analysis can correctly predict *Ube3a* genotype with high accuracy and that the behavioral severity score (PC1) is sensitive to treatment (77). Including USV testing in the *Ube3a*^*m*−*/p*+^ behavioral battery has the potential to increase the breadth of domains assessed and also increase the value of the battery by increasing the distance between WT mice and *Ube3a*^*m*−*/p*+^ littermates in principal component space. To test this hypothesis, we evaluated USVs and other behaviors within the same mice, and asked whether including USV data in multidimensional analysis would improve its utility.

## METHODS AND MATERIALS

### Animals

**Figure 1:**
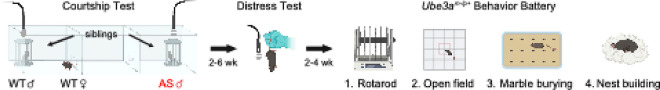
Longitudinal experimental design for vocalizations and behavior. The same cohort of mice (Cohort 1) went through three phases of behavioral testing: courtship, distress, and the *Ube3a*^*m-/p*+^ behavioral battery established by Sonzogni et al. (53). Weight (not shown) was recorded prior to rotarod testing. We recorded vocalizations during courtship and distress tests. Images created using Biorender.com.

**Figure 2:**
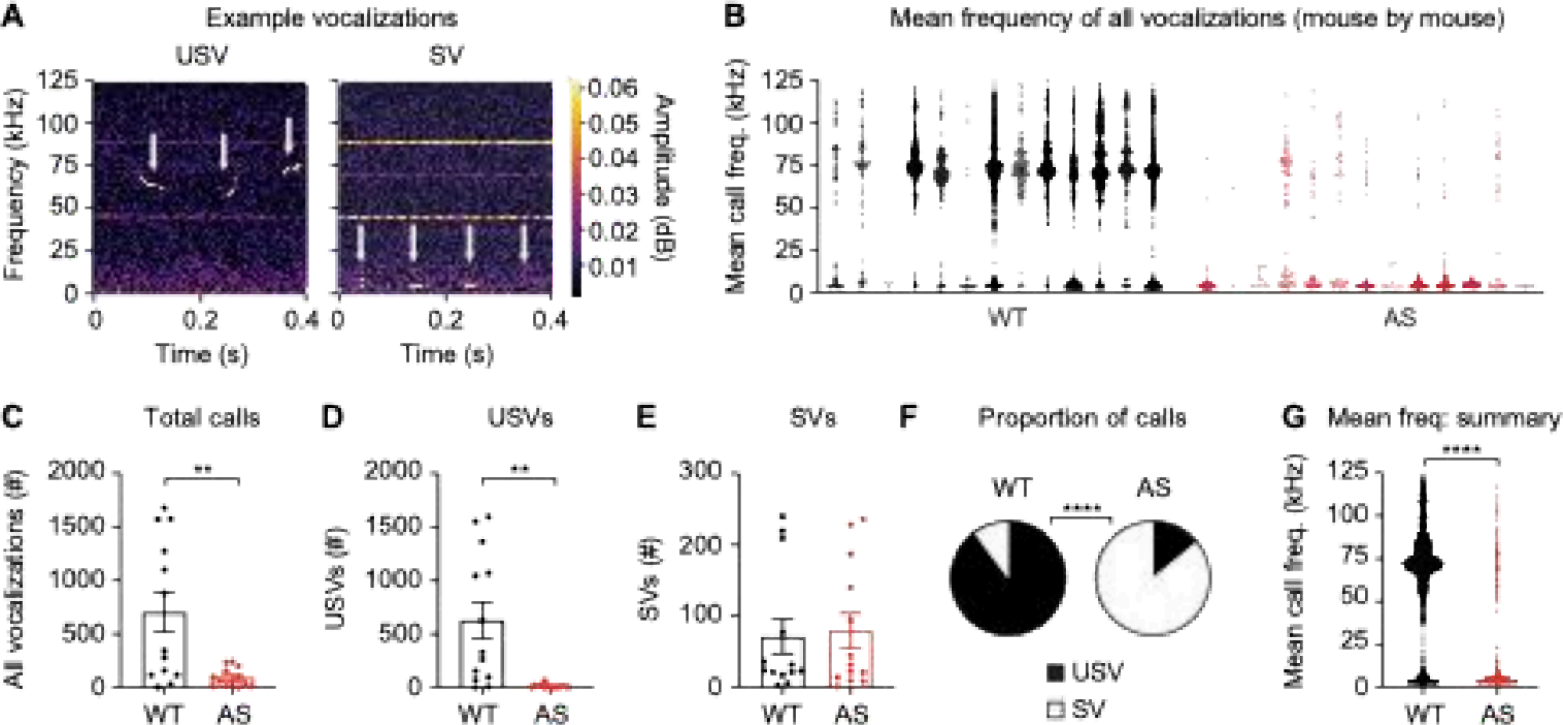
Ube3am−/p+ male mice make fewer USVs during courtship. (A) Representative examples of the two types of courtship vocalizations: USVs and SVs. White arrows indicate individual vocalizations. (B) Plot of the mean frequency of each vocalization made by males during the courtship test on a mouse-by-mouse basis. WT: *n* = 13 mice, *Ube3a*^*m*−*/p*+^ (AS): *n* = 13 mice. (C) *Ube3a*^*m*−*/p*+^ male mice made fewer total vocalizations during courtship. (D) *Ube3a*^*m*−*/p*+^ male mice made fewer USVs during courtship. (E) No genotype difference in the number of SVs produced. (F) *Ube3a*^*m-/p*+^ mice made a greater proportion of SVs. (G) *Ube3a*^*m*−*/p*+^ vocalizations had a lower mean frequency on a call-by-call basis (WT: *n* = 9096 calls, AS: *n* = 1206 calls). Data represent mean ± SEM; ****p < 0.0001; **p < 0.01; black: WT, red: *Ube3a*^*m*−*/p*+^ (AS).

All experimental protocols were approved by the Institutional Animal Care and Use Committee (IACUC) of Children’s National Hospital. All datasets used *Ube3a*^*m*−*/p*+^ mice and WT littermate controls (*Ube3a*^*m*+*/p*+^) on a congenic C57BL6/J background. Experimenters were blind to genotype during all testing and analysis. Experimental WT and *Ube3a*^*m*−*/p*+^ littermates were generated by crossing female *Ube3a*^*m*+*/p*−^ mice and male WT mice. The mice used in this study were grouped into one of three cohorts: a longitudinal cohort (Cohort 1) that underwent courtship, distress, and *Ube3a*^*m*−*/p*+^ behavior battery testing (Fig. 1) and two cohorts (Cohorts 2–3) that only underwent the distress test. Cohort 1 included 39 total adult mice aged P56–78 (WT male: *n* = 13, *Ube3a*^*m*−*/p*+^ male: *n* = 13, WT female stimulus: *n* = 13) and was used for the majority of analyses (Figs. 2–<link rid=“fig4”>4</link>, 5A–G, 6, S1-<link rid=“fig4”>4</link>, S6–7). Cohort 2, evaluating distress USVs in male and female adult mice, included 72 mice aged P51–126 (WT female: *n* = 16, WT male: *n* = 20, *Ube3a*^*m*−*/p*+^ female: *n* = 20, *Ube3a*^*m*−*/p*+^ male: *n* = 16) (Figs. 5H–I, S5). Cohort 3, evaluating distress USVs in aged mice, included 35 mice aged P304–345 (WT female: *n* = 11, WT male: *n* = 7, *Ube3a*^*m*−*/p*+^ female: *n* = 7, *Ube3a*^*m*−*/p*+^ male: *n* = 10) (Figs. 5J–K, S5).

### Behavioral testing

In Cohort 1, mice first performed the courtship test, then the distress test, and then the *Ube3a*^*m*−*/p*+^ behavior battery initially described by Sonzogni and colleagues (53) (weight, rotarod, open field, marble burying, and nest building tests) (Fig. 1). Time between the courtship and distress test was 2–6 weeks, and time between the distress test and the *Ube3a*^*m*−*/p*+^ behavior battery was 2–4 weeks. Methods for the *Ube3a*^*m*−*/p*+^ behavior battery were based on prior work (53, 77), but we did not perform a forced swim test. Cohorts 2–3 only underwent the distress test.

#### Courtship test.

We used methods based on a well-established three-chamber courtship paradigm that has been used in mice and other species (59, 78–81). This type of courtship paradigm, where one male is exposed to one female, has previously been shown to elicit vocalizations from male but not female mice (74, 82–85). WT and *Ube3a*^*m*−*/p*+^ male littermates were removed from the rest of their siblings and housed in dyads for at least five days prior to testing. Each WT female mouse was habituated separately in the center chamber (40 cm × 21 cm × 20 cm) of a three-chamber apparatus (40 cm × 68 cm × 20 cm) with both doors closed for 10 min and then with both doors open for an additional 10 min at least two hours prior to testing on the day of testing. Each stimulus female had to visit both of the side chambers in order to qualify for the courtship test, and all females qualified. After female habituation, the apparatus was thoroughly cleaned using 70% ethanol, and two male siblings (one WT, one *Ube3a*^*m*−*/p*+^) were randomly selected to be in either the left or right chamber. Each male mouse was placed in a round plexiglass cage (8 cm × 20 cm) with open bars around the sides. Males were habituated to that cage in the apparatus for 10 min. After the male habituation, the female stimulus mouse was placed into the center chamber of the three-chamber apparatus, the side chamber doors were opened, and behavior and vocalizations were recorded for 20 min. Behavior was recorded using a Kicteck HD1080P digital video camera, and USVs were recorded with ultrasonic microphones in each of the far chambers (see below for more details). We quantified the total time the female spent in each of the three chambers and nose-to-nose contact. Nose-to-nose contact was defined as the amount of time the female and male were face-to-face and less than a whisker’s distance apart. Behaviors were analyzed by manually scoring videos.

#### Distress test.

To elicit distress vocalizations, we used a modified tail suspension test (76). Mice were picked up by their tails from their home cage and immediately held approximately 2 inches below and 2 inches behind an ultrasonic microphone for 60 sec (Cohorts 1–2) or 30 sec (Cohort 3). After the recording, the mice were placed back in their home cages.

#### Rotarod.

Mice were placed on a rotating bar that accelerated from 4 to 40 rpm across 5 min at an acceleration rate of 7.2 rpm^2^ (Ugo Basile model #47600). Trials were complete once the mouse fell off, if three consecutive wrapping rotations were made, or if 5 min elapsed. Each day, the results of two trials with an inter-trial interval of one hour were averaged together. Experiments were run across five consecutive days with a two-day interval before open field testing.

#### Open field test.

Mice were placed into a 42 cm square open field arena (AccuScan Instruments, Inc., Columbus, OH) and were allowed to freely move for a single 10-min trial. The center square was defined as 21 cm × 21 cm. The data was collected using the open field activity monitoring system (Omnitech Electronics, Inc. SuperFlex Open Field System) which uses photocell emitters and receptors forming an x–y grid of infrared beams. Total distance moved and time spent in the center square were recorded using infrared beam break information. The experiment was run on one day with a three-day interval before marble burying testing.

#### Marble burying.

Mice were placed individually in a 16 × 8 in cage with ~ 4 cm of bedding (Bed-o’Cobs 1/4” bedding) and 20 black glass marbles arranged in a 5 × 4 array for a single 30 min trial. A marble was considered buried if it was > 50% covered with bedding at the end of the trial.

#### Nest building.

Immediately after marble burying, mice were habituated to single housing as well as new nesting material (Bio-Rad 7.5 × 10 cm extra thick block filter paper; 11 ± 1 g) for 4 days prior to testing. During testing, new nesting material was introduced on day 1 and unused material was weighed daily across five days.

### Ultrasonic vocalization recording

Vocalizations were collected using UltraSoundGate CM16/CMPA series microphones capable of detecting broadband sound (2–250 kHz). Microphone channels were calibrated to equal gain (− 60 dB noise floor) using the Avisoft Bioacoustics RECORDER program. The RECORDER software produced .wav file recordings that we visualized using SASLAB Pro (Avisoft Bioacoustics). Recordings were collected at a 250 kHz sampling rate with a 16-bit resolution. In the courtship test, microphones were placed approximately two inches above each cage containing the male mice. In the distress test, microphones were placed approximately 2 inches above and 2 inches in front of the mouse being held by its tail.

### Vocalization analysis

We used DeepSqueak (version 2.7.0) to visualize the spectrograms of each audio file (86). Once DeepSqueak converted the original .wav files into corresponding sonograms, we manually excluded noise, and detected vocalizations manually using the DeepSqueak user interface. We categorized social courtship vocalizations into two types: syllable vocalizations (SVs) in the audible 5–15 kHz range (87, 88) and ultrasonic vocalizations (USVs) in the 25–130 kHz range (89–91). We found no vocalizations in the 15–25 kHz boundary during courtship testing. Because we have two male mice in the chamber, we also wanted to ensure that USVs recorded from the right microphone were excluded from analysis if their origin appeared to be from the left chamber and vice versa. To accomplish this goal, we manually evaluated any calls that overlapped on the left and right microphones within 0.2 seconds. We viewed left and right spectrograms side-by-side to determine if the USVs had the same spectral properties and were likely the same USV being picked up on the far microphone (see **Additional File 1: Supplementary Fig. 1** for an example). Calls picked up on both microphones were identified and assigned to the microphone where the call was louder. The far microphone picked up less than three percent of USVs (**Additional File 1: Supplementary Fig. 1**) .

Using an established system for categorization, we categorized courtship USVs as either simple or complex based on the properties of calls (91, 92). Simple USVs are defined as USVs with no inflection points that either increase frequency with time (positive slope) or decrease frequency with time (negative slope). We calculated the ratio of simple to complex USVs on a per call level, rather than a per-mouse level. To quantify the slope of simple USVs, we first calculated the absolute value of the slope for all USVs, regardless of direction. We then separately quantified the slopes of calls with a positive slope and those with a negative slope and computed the ratio of positive to negative slopes (+/− ratio) (93). Complex USVs are defined as having at least one inflection point but may have many inflection points. For the distress test, we categorized calls as either USVs (25–130 kHz range), squeaks (5–20 kHz), or ranging vocalizations that span both the audible and USV frequency ranges (5–130 kHz) (93). We calculated the ratio of all distress calls on a per call level, rather than a per-mouse level. The reason for quantifying call type ratios on a per call level is to avoid over-weighting the impact of a mouse that makes e.g. only one or two USVs (Fig. 2b).

**Figure 3:**
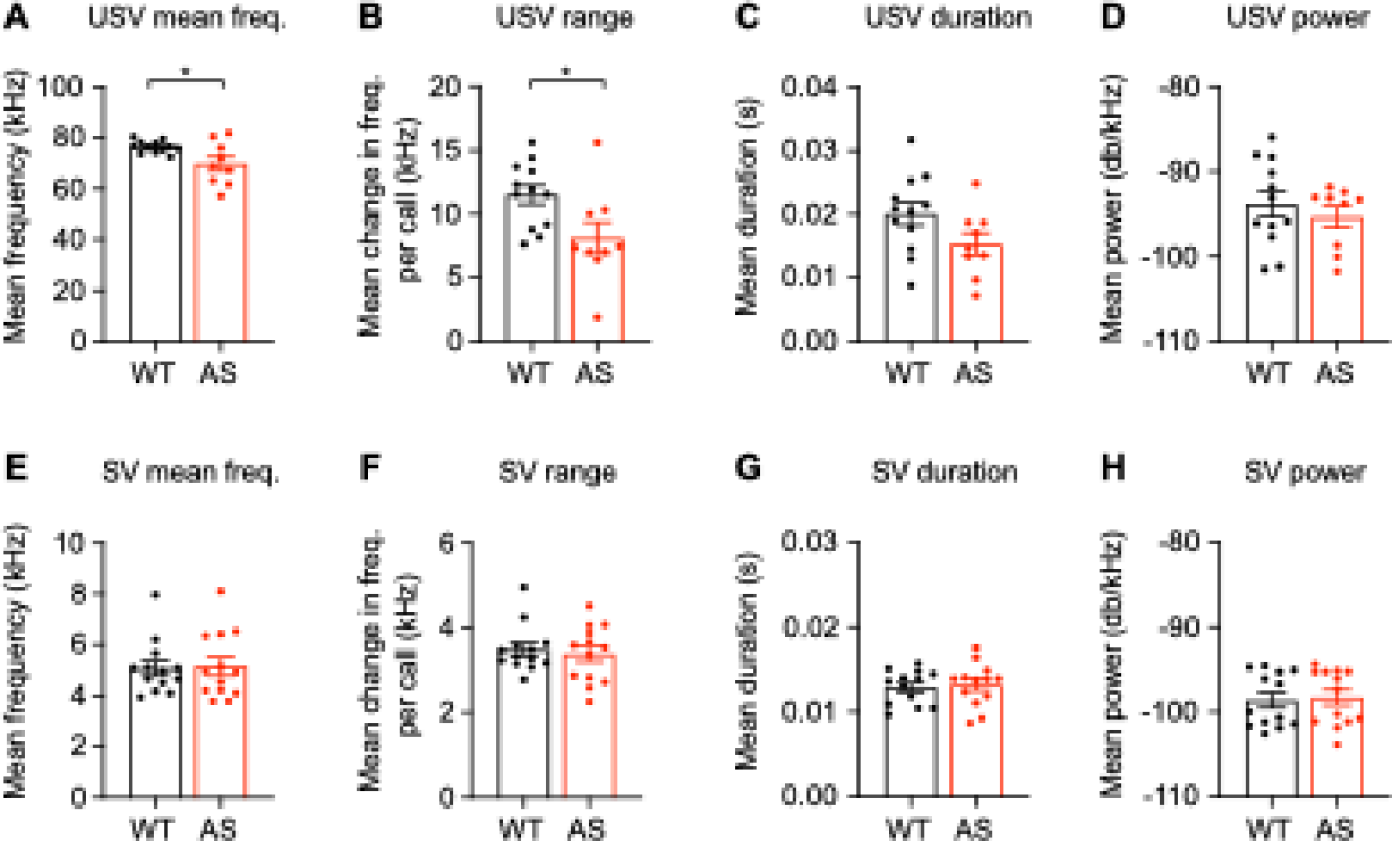
Spectral properties of USVs but not SVs are different during courtship. *Ube3a*^*m*−*/p*+^ male mouse USVs during courtship have (A) a higher mean frequency and (B) a smaller mean frequency range, but not difference in (C) mean duration or (D) mean power (WT: *n* = 12, AS: *n* = 9). *Ube3a*^*m*−*/p*+^ male mouse SVs during courtship have no difference in (E) mean frequency, (F) mean frequency range, (G) duration, or (H) power of SVs (WT: *n* = 13 mice, AS: *n* = 13 mice). The n is different for USVs and SVs because one WT mouse and four *Ube3a*^*m*−*/p*+^ mice did not make USVs. Data represent mean ± SEM; *p<0.05; black: WT, red: *Ube3a*^*m*−*/p*+^ (AS).

**Figure 4:**
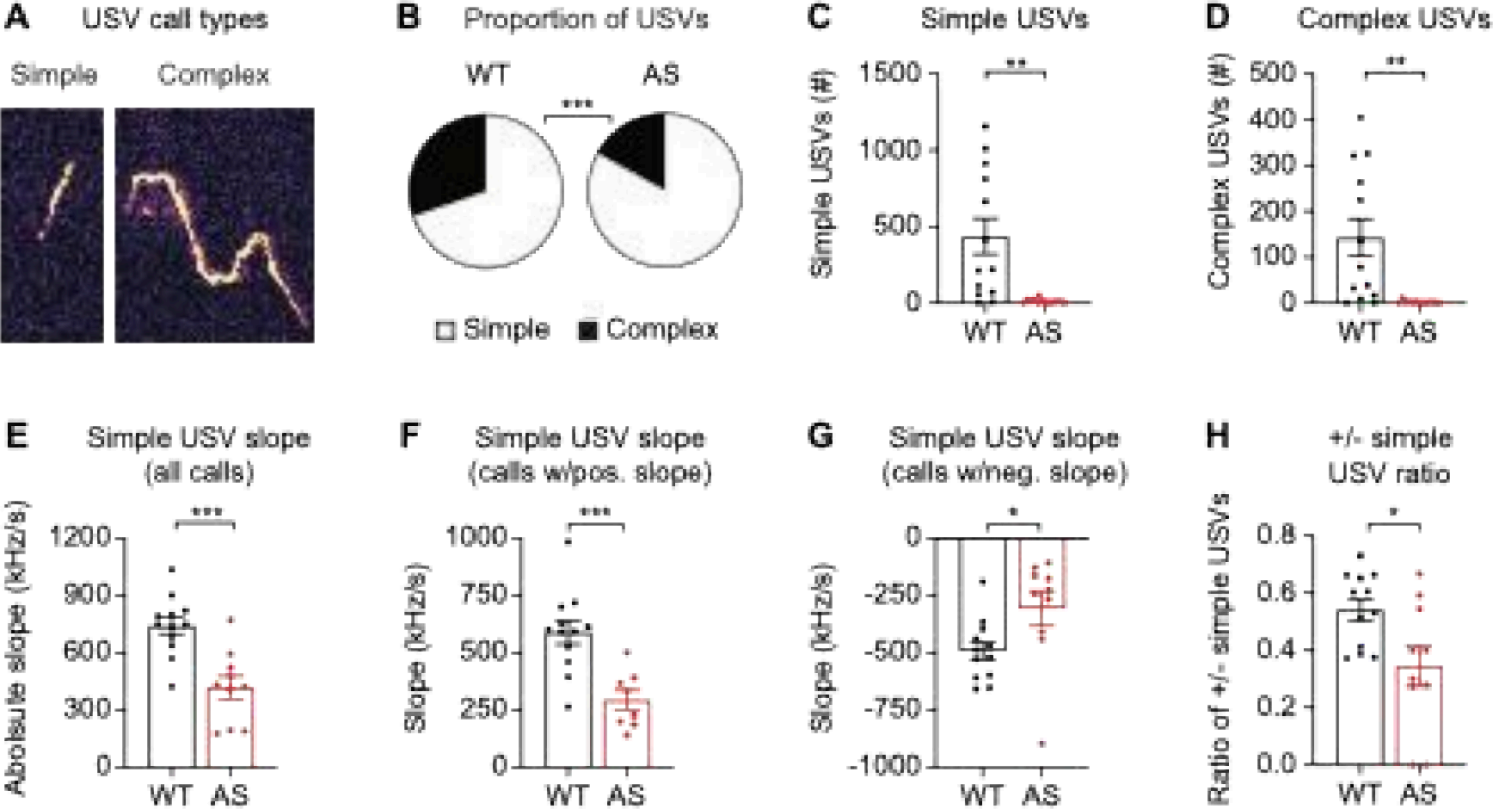
Spectral properties of Ube3am−/p+ male courtship USVs are less complex. (A) Representative examples of simple and complex courtship USVs. Simple calls have no inflection points and complex calls have one or more inflection point. (B) *Ube3a*^*m*−*/p*+^ (AS) mice made a greater proportion of simple USVs (WT: *n* = 9,098 calls, AS: *n* = 1,207 calls) than WT littermates. *Ube3a*^*m*−*/p*+^ mice made fewer total (C) simple and (D) complex USVs. The slope of simple USVs is less steep in *Ube3a*^*m*−*/p*+^ mice when quantified across (E) all simple calls (WT: *n* = 12, AS: *n* = 10), (F) simple calls with a positive slope (WT: *n* = 12, AS: *n* = 8), and (G) simple calls with a negative slope (WT: *n* = 12, AS: *n* = 10). (H) The ratio of positive to negative sloped simple USVs was smaller for *Ube3a*^*m*−*/p*+^ mice. Data represent mean ± SEM; ***p < 0.001; **p < 0.01; *p < 0.05; black: WT, red: *Ube3a*^*m-/p*+^ (AS).

**Figure 5:**
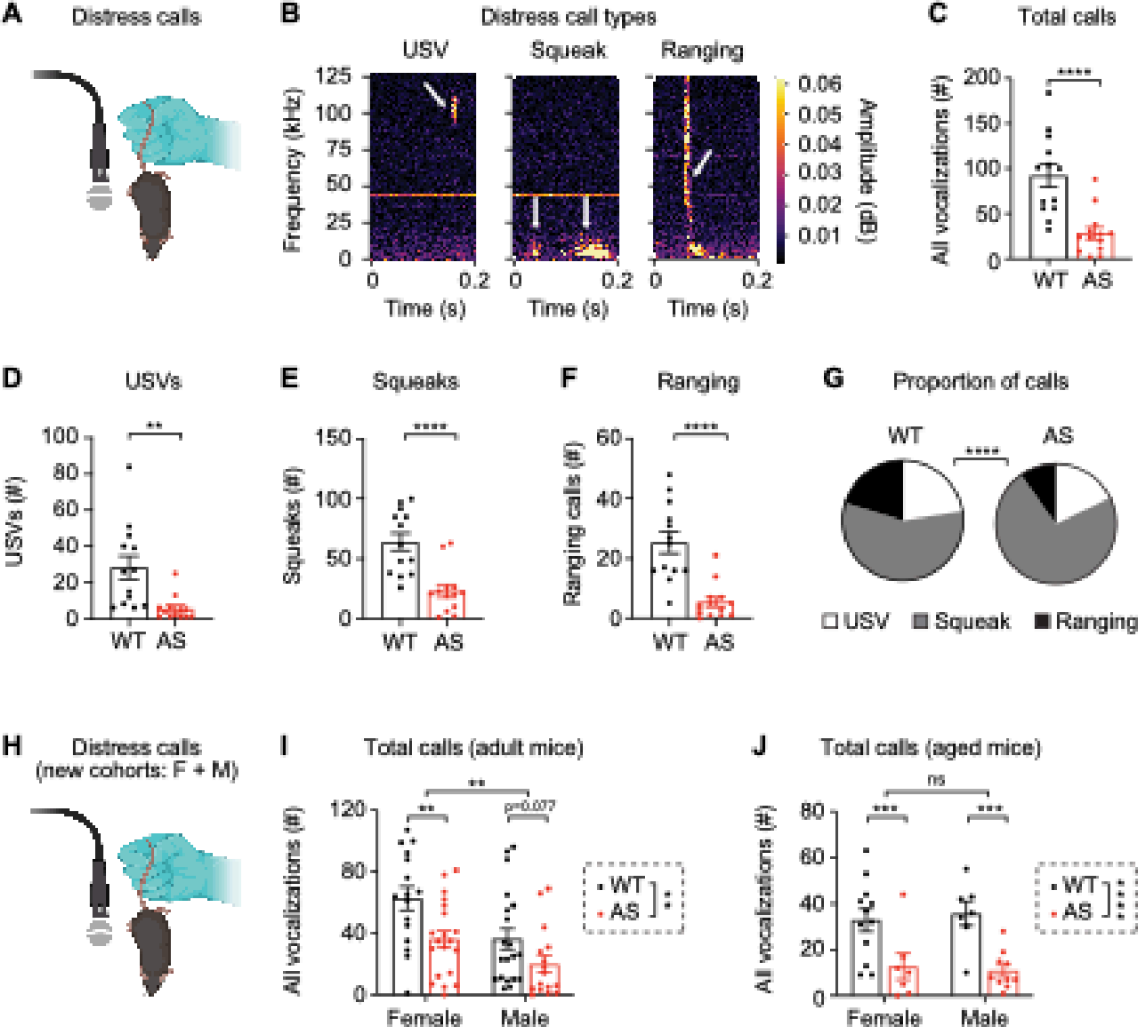
Ube3am−/p+ mice make fewer distress USVs. (A) Distress testing via tail suspension in same cohort of male mice that performed courtship testing (Cohort 1). (B) Representative example vocalizations of the three types of distress vocalizations: USVs, squeaks, and ranging USVs. White arrows indicate individual vocalizations. (C) *Ube3a*^*m*−*/p*+^ (AS) mice made fewer total vocalizations during distress (WT: *n* = 13 mice, AS: *n* = 13 mice). *Ube3a*^*m*−*/p*+^ mice also made fewer (D) USVs, (E) squeaks, and (F) ranging calls. (G) *Ube3a*^*m*−*/p*+^ mice made a greater proportion of low-frequency squeaks WT: *n* = 1,628 calls, AS: *n* = 398 calls). (H) Distress testing in two cohorts including male and female littermates: adult mice and aged mice. (I) Total distress calls by genotype in sex-balanced adult cohort (WT: *n* = 36 mice, AS: *n* = 36 mice). (J) Total distress calls by genotype in sex-balanced aged cohort (WT: *n* = 18 mice, AS: *n* = 17 mice). Data represent mean ± SEM; ****p < 0.0001; ***p<0.001; **p<0.01; black: WT, red: *Ube3a*^*m*−*/p*+^ (AS). Images in panels A and H created using Biorender.com.

**Figure 6:**
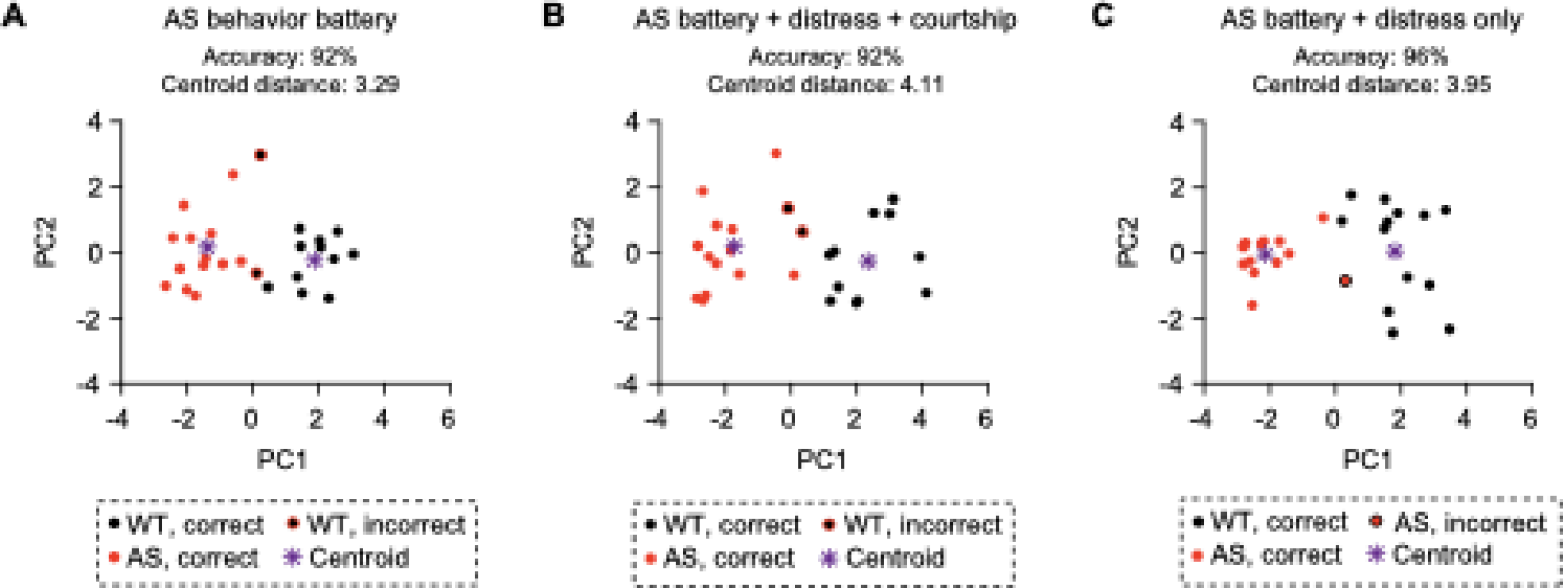
Multidimensional analysis that includes USVs predicts genotypes accurately. (A) Multidimensional analysis using data from the *Ube3a*^*m*−*/p*+^ behavior battery demonstrates accurate clustering of animals in principal component space. (B) Multidimensional analysis using data from the *Ube3a*^*m*−*/p*+^ behavior battery and vocalization data from courtship and distress tests. Accuracy remains high, and centroid distance increases. (C) Multidimensional analysis using data from the *Ube3a*^*m*−*/p*+^ behavior battery and vocalization data from the distress test only. Centroids and centroid distance are calculated based on actual genotypes, not *k*-means clusters.

In both courtship and distress tests, this study quantified four main characteristics of vocalizations: mean frequency (kHz), frequency range (kHz), duration (ms), and power (dB/kHz). DeepSqueak automatically calculates these parameters based on user-identified calls (86). Mean frequency is calculated by finding the point of the call with the lowest and highest frequency and taking the average. Range is calculated by summing the distance between the lowest and highest frequency points of the call. Duration is the length of a call across time. Power calculates how loud the call is relative to its frequency, and is defined as the discrete Fourier transform of the signal X(f) in this formula, P=∑Xf2f. We primarily analyzed vocalizations by averaging call properties on a mouse-by-mouse basis (Figs. 2C–E, 3A–H, 4B–H, 5C–F, 5H–J, 6A–C, S2C, S2E, S3A-B, S4A-L, S5B-D, S5H-J, S6A-H, S 7A-B) but also additionally analyzed some vocalizations on a call-by-call basis where noted (Figs. 2B, 2F–G, S2A-B, S2D, 5G, S5E-F, S5K-L).

### Multidimensional analysis of mouse behavior

We used the MATLAB graphical user interface PUMBAA (github.com/sidorovlab/PUMBAA) to perform multidimensional analysis of behavior and vocalization properties (77). Multidimensional analysis using PUMBAA consists of a series of steps: data selection, standardization, principal component analysis (PCA), k-means clustering, and validation, as described in our prior work (77). *Data selection*: For the *Ube3a*^*m*−*/p*+^ behavior battery, seven total measures were included: weight, rotarod day 1, rotarod day 5, open field distance traveled, open field center time, marbles buried, and nest building at day 5 (Fig. 6A). Redundant measures (e.g., intermediate, non-independent time points for rotarod and nest building) were excluded *a priori* from multidimensional analysis. For the *Ube3a*^*m*−*/p*+^ behavior battery plus distress and courtship calls (Fig. 6B), 12 total measures were included: *Ube3a*^*m*−*/p*+^ behavior battery (seven measures as above), distress calls (number of USVs, number of squeaks, number of ranging USVs), and courtship calls (number of USVs and number of SVs). For the *Ube3a*^*m*−*/p*+^ behavior battery plus distress calls only (Fig. 6C), nine total measures were included in the multidimensional analysis: *Ube3a*^*m*−*/p*+^ behavior battery (six measures as above) and distress calls (three measures as above). *Standardization*: All measures were standardized using a z-score (z = (data point − group means)/standard deviation) to account for different units across tests. In prior work, we standardized male and female data separately for tests where there was a sex difference (77), but that was not relevant here because only male mice were used for this analysis. *Principal component analysis*: We performed PCA using the pca() function in MATLAB and calculated the amount of variance explained by each PC using a Scree plot and the loading distribution of principal components using the coefficient outputs from PCA (**Additional File 7**: **Supplementary Fig. 7**). *k-means clustering*: k-means clustering was performed in principal component space using the kmeans() function in MATLAB with k = 2 clusters. *Validation*: We compared the actual genotypes of animals to their assigned cluster and calculated the percentage correct. We also calculated the centroids of genotype-based groups by taking the mean of all x and y positions for each mouse in principal component space. Here, centroids were calculated based on actual genotype and not k-means cluster. The distance between WT and *Ube3a*^*m*−*/p*+^ centroids was defined as the absolute value of the square root of ${\left(x_{1}-x_{2}\right)}^{2}+{\left(y_{1}-y_{2}\right)}^{2}$, where x and y represent the coordinates of centroids defined by genotype in 2PC space.

### Statistics

Statistical analysis was performed using GraphPad Prism 9 and MATLAB R2023a (Mathworks). We used unpaired t-tests for the majority of comparisons between WT and *Ube3a*^*m*−*/p*+^ mice when assessing vocal production and behavior (Figs. 2C–E, 2G, 3A–H, 4C–H, 5C–F, S4A-L, S6A, S6C-H). In the courtship test, we used a one-way repeated measures (RM) ANOVA with a Sidak *post hoc* test when quantifying where the WT female spent time in the three-chamber arena () and a paired t-test when comparing the time she spent nose-to-nose with each male (). When assessing the effects of genotype and sex in Cohorts 2 and 3, we used a two-way ANOVA (Figs. 5I–J, S5B-D, S5H-J) and *post hoc* Fisher’s LSD tests when there was a statistically significant main effect of genotype or sex. When assessing the effects of time (within animals) and genotype, we used a two-way RM ANOVA and *post hoc* Fisher’s LSD tests when there was a statistically significant main effect of genotype or time (**Figs. S2C, S2E, S6B**). When comparing whether two proportions differed, we used a chi-squared Fisher’s exact test (Figs. 2F, 4B, 5G, S5E-F, S5L-M). For all figures, data are represented as mean +/− SEM and *p < 0.05, **p < 0.01, ***p < 0.001, and ****p < 0.0001.

## RESULTS

### Ube3a ^m−/p+^ male mice make fewer USVs during courtship

To assess vocalizations during courtship, we tested WT and *Ube3a*^*m*−*/p*+^ male sibling dyads in a courtship paradigm (Fig. 1). We placed male siblings (one WT, one *Ube3a*^*m*−*/p*+^) on the far ends of a three-chambered apparatus, introduced an unrelated WT female stimulus mouse, and recorded vocalizations and behavior for 20 minutes. We observed and quantified two categories of vocalizations: ultrasonic vocalizations (USVs) above 25 kHz and syllable vocalizations (SVs) in the 5–15 kHz range (Fig. 2A–B). *Ube3a*^*m*−*/p*+^ male mice made fewer total calls than WT littermates (Fig. 2C; t^(24)^ = 3.31, *p* = 0.0030, unpaired t-test), including significantly fewer total USVs (Fig. 2D; t_(24)_ = 3.61, *p* = 0.0014) but no difference in the number of SVs (Fig. 2E; t_(24)_ = 0.245, *p* = 0.81). As a result, WT mice made a higher proportion of USVs than *Ube3a*^*m*−*/p*+^ mice (Fig. 2F; *p* < 0.0001, Fisher’s exact test) and the average frequency of all vocalizations was lower in *Ube3a*^*m*−*/p*+^ males relative to WT littermates (Fig. 2G; t_(10300)_ = 70.11, *p* < 0.0001). Male mice produced USVs more frequently in the last ten minutes of the test compared to the first ten minutes, and the genotype difference in USVs was more pronounced in the last ten minutes (**Additional File 2: Supplementary Fig. 2**). Surprisingly, despite differences in USV production between WT and *Ube3a*^*m*−*/p*+^ males, WT females did not show a behavioral preference for either male **(Additional File 3: Supplementary Fig. 3).**

### Spectral properties of USVs but not SVs are different in Ube3a^m−/p+^ male mice during courtship

Previous work found changes in the spectral properties of adult female *Ube3a*^*m*−*/p*+^ USVs during female-female social interactions (42). Therefore, we asked whether the spectral properties of courtship vocalizations differed between WT and *Ube3a*^*m*−*/p*+^ male mice during courtship. Because the proportion of USVs:SVs was different between WT and *Ube3a*^*m*−*/p*+^ mice, and because USVs and SVs have very different spectral properties, we analyzed each vocalization type separately. *Ube3a*^*m*−*/p*+^ mice produced courtship USVs with a lower mean frequency (Fig. 3A; t_(19)_ = 2.58, *p* = 0.018, unpaired t-test) and a shorter range (Fig. 3B; t_(19)_ = 2.48, *p* = 0.023) but no difference in duration (Fig. 3C; t(19) = 1.870, *p* = 0.077) or power (Fig. 3D; t_(19)_ = 0.733, *p* = 0.473) relative to WT littermates. Conversely, there were no differences in the properties of SVs between WT and *Ube3a*^*m*−*/p*+^ mice (mean frequency: Fig. 3E; t_(24)_ = 0.15, *p* = 0.88; range: Fig. 3F; t_(24)_ = 0.44, *p* = 0.66; duration: Fig. 3G; t_(24)_ = 0.33, *p* = 0.74; power: Fig. 3H; t_(24)_ = 0.25, *p* = 0.81).

### Ube3a ^m−/p+^ male mice make fewer complex USVs

Complexity is a critical property of male courtship USVs. Complex USVs – operationally defined as calls with at least one inflection point (94) – take more energy to produce and are honest signals of healthier mice (83, 95, 96). Previous work in *Ube3a*^*m*−*/p*+^ mice found differences in the loudness and duration of complex calls but no difference in the number of complex calls during a female-female social interaction test (42). In courtship settings, male mice preferentially use complex USVs to attract mates (59); thus, we asked whether male *Ube3a*^*m*−*/p*+^ mice are impaired in their ability to make complex USVs. To do this, we categorized courtship calls by type: simple USVs have no inflection point and complex USVs have at least one inflection point (94) (Fig. 4A). During courtship, *Ube3a*^*m*−*/p*+^ male mice produced a smaller proportion of complex USVs compared to WT mice (Fig. 4B; *p* = 0.0001, Fisher’s exact test). In absolute terms, *Ube3a*^*m*−*/p*+^ mice produced fewer calls of both types: simple (Fig. 4C; t_(24)_ = 3.64, *p* = 0.0012, unpaired t-test) and complex (Fig. 4D; t_(24)_ = 3.47, *p* = 0.0020). The slope of simple USVs is also thought to be meaningful: steeper calls have been linked to social interaction (97, 98) and prior sexual experience (99, 100). In addition, the steepness of simple calls is positively correlated with the number of complex calls within animals (101, 102). Simple USVs from *Ube3a*^*m*−*/p*+^ mice had less steep slopes than WT littermates (Fig. 4E; t_(20)_ = 4.36, *p* = 0.0003). Simple calls were less steep both in calls with positive slopes (Fig. 4F; t_(18)_ = 4.05, *p* = 0.0008) and calls with negative slopes (Fig. 4G; t_(20)_ = 2.34, *p* = 0.030). Simple calls from *Ube3a*^*m*−*/p*+^ mice were also more likely to have a negative slope (Fig. 4H; t_(20)_ = 2.55, *p* = 0.019). Overall, the complexity of courtship USVs is greatly diminished in *Ube3a*^*m*−*/p*+^ mice: they make fewer complex calls with inflection points, and their simple calls have flatter slopes.

### Ube3a ^m−/p+^ mice make fewer distress USVs

In addition to quantifying directed social vocalizations during courtship, we also evaluated the properties of undirected, nonsocial distress vocalizations induced by tail suspension in the same male mice 2–6 weeks after courtship testing (Fig. 1, Fig. 5A). We categorized three types of distress calls based on their frequency range (103, 104): USVs that occur in the ultrasonic frequency range only (above 50 kHz), squeaks that are in the audible range (below 25 kHz), and ranging vocalizations that span both USV and audible ranges (5–120 kHz) (Fig. 5B). *Ube3a*^*m*−*/p*+^ mice made fewer overall distress calls (Fig. 5C; t_(24)_ = 4.49, *p* = 0.0002), including fewer USVs (Fig. 5D; t_(24)_ = 3.39, *p* = 0.002), fewer squeaks (Fig. 5E; t_(24)_ = 4.53, *p* < 0.0001), and fewer ranging vocalizations (Fig. 5F; t_(24)_ = 5.03, *p* < 0.0001) than WT littermates. The proportion of call types differed by genotype (Fig. 5G; *p* < 0.0001, Fisher’s exact test), as *Ube3a*^*m*−*/p*+^ mice had a higher percentage of low-frequency squeaks. The properties of distress calls (categorized by type) were generally not different by genotype, with the exception that the mean frequency of USVs was decreased in *Ube3a*^*m*−*/p*+^ mice **(Additional File 4: Supplementary Fig. 4).**

Because both female and male mice readily produce distress USVs (75), we next asked whether the dysregulation of distress USVs in *Ube3a*^*m*−*/p*+^ male mice is sex-specific. In a new cohort (Cohort 2) including adult female and male littermates (Fig. 5H), *Ube3a*^*m*−*/p*+^ mice made fewer calls overall than WT littermates (Fig. 5I; two-way ANOVA, main effect of genotype: F(1, 68) = 11.07, *p* = 0.0010). The difference in calls between WT and *Ube3a*^*m*−*/p*+^ mice was statistically significant for females (Fig. 5I; *p* = 0.005, *post hoc* uncorrected Fisher’s LSD) but not statistically significant in males in this cohort (Fig. 5I; *p* = 0.077). Females overall made more distress calls than males (Fig. 5I; main effect of sex: F_(1, 68)_ = 10.87, *p* = 0.0016), but there was not a significant genotype by sex interaction (Fig. 5I; F_(1, 68)_ = 0.615, *p* = 0.44). In this sex-balanced cohort, *Ube3a*^*m*−*/p*+^ mice made fewer distress calls of all types (USV, squeak, ranging) and the proportion of call types was different by genotype (**Additional File 5: Supplementary Fig. 5A-F).** Because C57BL/6 mice develop substantial hearing loss as they age (105), we then asked whether vocal feedback impacted vocal production by quantifying distress calls in a cohort of aged (P300–350) mice (Cohort 3). Aged male and female *Ube3a*^*m*−*/p*+^ mice made fewer overall calls than WT littermates (Fig. 5J; two-way ANOVA, main effect of genotype: F(1, 32) = 21.85, *p* < 0.0001). The difference in overall calls was significant for both females and males (Fig. 5J; Female WT vs. AS: *p* = 0.0098, *post hoc* uncorrected Fisher’s LSD; Male WT vs. AS: *p* = 0.0022). Aged *Ube3a*^*m*−*/p*+^ mice made fewer distress calls of all types (USV, squeak, ranging) and the proportion of call types differed by genotype **(Additional File 5: Supplementary Fig. 5G-L).**

### USVs can be included in the established Ube3a^m−/p+^ behavior battery

Because USVs were consistently reduced in *Ube3a*^*m*−*/p*+^ mice across courtship and distress settings, we next evaluated the utility of including USV tests in the existing *Ube3a*^*m*−*/p*+^ behavioral battery that is widely used to evaluate response to treatment (53). In the same male mouse cohort that performed courtship and distress testing, we also evaluated performance on a battery including weight, rotarod, open field, marble burying, and nest building. We then used multidimensional analysis to quantify overall behavioral severity in principal component space (77). As expected, multidimensional analysis of behavioral data enabled clustering of WT and *Ube3a*^*m*−*/p*+^ genotypes with high accuracy: 92% (24/26) of genotypes were predicted correctly based on behavior alone (Fig. 6A). This result was not surprising, as *Ube3a*^*m*−*/p*+^ mice showed statistically significant impairments on each individual assessment **(Additional File 6: Supplementary Fig. 6).** Next, we wanted to determine how adding vocalizations to multidimensional analysis influenced clustering and accuracy. Including courtship and distress vocalization data in multidimensional analysis maintained 92% accuracy and increased the distance between the centroids of WT and *Ube3a*^*m*−*/p*+^ clusters from 3.29 to 4.11 units in PC space (Fig. 6B). Including distress vocalizations alone resulted in 96% accuracy and a centroid distance of 3.95 units (Fig. 6C). Across models, PC1 accounted for ~ 50% of variance in the dataset and most behaviors loaded onto PC1 **(Additional File 7: Supplementary Fig. 7).** Overall, our results demonstrate that it is feasible to include vocalization data in multidimensional analysis and this approach may increase the distance between WT and *Ube3a*^*m*−*/p*+^ mice in principal component space.

## DISCUSSION

The goal of this study was to evaluate vocalizations in adult *Ube3a*^*m*−*/p*+^ mice during social courtship and nonsocial distress settings. During social courtship, USVs were decreased nearly 50-fold in *Ube3a*^*m*−*/p*+^ male mice relative to WT male littermates, despite normal SV production in *Ube3a*^*m*−*/p*+^ males (Fig. 2). In the nonsocial distress test, female and male *Ube3a*^*m*−*/p*+^ mice made fewer vocalizations of all types (Fig. 5). Our work is generally consistent with prior findings of decreased USVs in adult female *Ube3a*^*m*−*/p*+^ mice during a female-female social interaction task (42). Together, our results and prior findings suggest that abnormal vocalizations are a consistent feature of *Ube3a*^*m*−*/p*+^ mice that generalize across both social and nonsocial contexts. Because impaired USVs generalize across behavioral contexts, our results support the hypothesis (42) that these impairments may be driven by a generalized motor dysfunction in the production of calls, and not by context-specific social impairments.

In addition to observing a decreased number of calls, we found that several properties of courtship vocalizations were meaningfully altered in *Ube3a*^*m*−*/p*+^ mice. First, courtship USVs had lower mean frequencies and shorter ranges (Fig. 3). Moreover, *Ube3a*^*m*−*/p*+^ mice made a smaller proportion of complex USVs relative to WT mice (Fig. 4). We also found structural differences in simple USVs—*Ube3a*^*m*−*/p*+^ male mice made USVs with flatter slopes (Fig. 4). Changes to these vocal characteristics may indicate that *Ube3a*^*m*−*/p*+^ mice have difficulty producing more challenging and energetically taxing vocalizations (106). More re ned USV structure is associated with more developmentally mature mice (102) and is characteristic of courtship (59). For example, during courtship, males progressively make USVs with steeper negative slopes (107) and higher frequency vocalizations are preferred by females (108). Thus, our results suggest that structural impairments in *Ube3a*^*m*−*/p*+^ USV production may be behaviorally meaningful in the context of courtship. Future work is needed to understand if and how impairments in courtship USVs might affect courtship itself in *Ube3a*^*m*−*/p*+^ mice. Unlike social courtship vocalizations, we found that the structure of nonsocial distress vocalizations was generally less impaired in *Ube3a*^*m*−*/p*+^ mice; distress USVs had a lower mean frequency, but all other properties were normal **(Additional File 4: Supplementary Fig. 4).** Thus, an advantage of courtship USV testing over distress testing is the ability to quantify subtle differences in USV properties/structure in addition to the number of calls.

The directionality of USV slope (i.e. positive or negative) is a property that may be behaviorally meaningful and provide insights into the nature of USV impairments in *Ube3a* mutants. During courtship, we found that simple USVs produced by *Ube3a*^*m*−*/p*+^ males are less likely than WT littermates to have a positive slope (Fig. 4H). The directionality of USV slope is important because it has been linked to motor coordination during breathing in mice (109). Most mouse vocalizations occur during exhalation, and cardinal breathing muscle activity can alter the vocalization slope (106, 109, 110). When exposed to female urine, male WT mice produced both negative- and positive-slope USVs: negative-slope USVs occurred during normal exhalation, but nearly all positive-slope USVs during “mini-breaths” contracting their cardinal breathing muscle (109). This may be behaviorally meaningful because positive-slope USVs are associated with greater positive affect and social (vs. nonsocial) interactions in large home cage arenas with different sensory areas (97, 111, 112). Because we saw a decrease in the proportion of positive-slope USVs in *Ube3a*^*m*−*/p*+^ mice, it could suggest difficulty activating their cardinal breathing muscle during expiration and may broadly contribute to social deficits.

More broadly, our work supports prior findings that vocalizations are disrupted in *Ube3a*^*m*−*/p*+^ rodent models across many behavioral settings. The first studies on vocalizations in *Ube3a*^*m*−*/p*+^ mice were done in pups using the pup isolation paradigm, where pups separated from their mother emit social distress calls to elicit retrieval back to the nest (113, 114). *Ube3a*^*m*−*/p*+^ mouse pups make fewer vocalizations around postnatal days 7–10 compared to WT littermates and more vocalizations around postnatal days 13–15 compared to WT littermates (71, 72). Abnormal pup calls were also reported in *Ube3a*^*m*−*/p*+^ rats (115). While pup isolation provides valuable insights into vocalization system development, phenotypes in *Ube3a*^*m*−*/p*+^ mice are extremely sensitive to the age of pups. In contrast, we found that decreased distress calls are a stable and persistent feature of *Ube3a*^*m*−*/p*+^ adults, observed in cohorts ranging from ~ P50-P130 to ~ P300-P350 (Fig. 5). Thus, USV testing in adults may be better suited for evaluating the effect of treatment. Perrino et al. (42) comprehensively analyzed *Ube3a*^*m*−*/p*+^ USVs during adult female-female social interactions: *Ube3a*^*m*−*/p*+^ females spent less time vocalizing, made fewer total USVs, fewer at and short USVs, and had louder “noisy” and “complex” USVs. In addition, call number and complexity correlated with motor performance on other tests such as the rotarod (42). Another study found that during a brief, 5-minute male-female social interaction with older mice (12-month-old), WT but not *Ube3a*^*m*−*/p*+^ mice vocalized (116). However, USV phenotypes in *Ube3a*^*m*−*/p*+^ mice may be sensitive to background strain: our work and the studies above used mice on a C57BL/6 background, whereas other groups have reported conflicting results with an FVB background (117). The *Ube3a*^*m*−*/p*+^ rat model of AS has also enabled quantification of USVs in other settings such as juvenile social play (118).

Our data suggest that including USV assessments in *Ube3a*^*m*−*/p*+^ behavioral testing is feasible, robust, and adds value to the widely used battery (53). The decreased number of distress calls was extremely robust in *Ube3a* mutants, observed in three separate cohorts (males only, females and males, aged females and males; Fig. 5). The widely used *Ube3a*^*m*−*/p*+^ behavioral battery (53) does not model impaired communication, which is one of the most severe, ubiquitous, and long-lasting phenotypes associated with AS, so including USV testing would increase the breadth of phenotypes modeled. In addition to improving the face validity of the behavioral battery, including USVs may improve our ability to separate mouse genotypes in principal component space based on overall behavior (Fig. 6). Our prior work demonstrated that PC1 can be used as an overall index of behavioral severity that is sensitive to treatment (77). Here, we show that including courtship and distress vocalization data in principal component analysis increases the distance between WT and *Ube3a*^*m*−*/p*+^ centroids in principal component space, driven mainly by a change in PC1. Interestingly, including only distress but not courtship data is nearly as effective, suggesting that distress vocalizations alone can improve the ability of the *Ube3a*^*m*−*/p*+^ battery to model impaired behavior in mice. This finding is important because a single session of distress testing is feasible at any age in adult mice and can be performed at any point of a longer behavior battery, unlike male-female, female-female, and pup testing, which is more time-consuming and potentially disruptive. However, distress testing may not be best suited for evaluating the ability to produce complex calls, and less is known about the neural circuitry of distress calls compared to courtship (119, 120). Overall, the benefits of distress vocalizations make this test an ideal candidate to include in existing behavior batteries for preclinical testing despite some its limitations relative to courtship, female-female social, or pup USV tests.

Because USV testing in mice is especially sensitive to protocol, it is important to carefully consider several key methodological parameters, particularly for courtship testing. First, C57BL/6 mice are notoriously quiet in novel situations relative to other strains, and it can be challenging to reliably elicit vocalizations during a short testing window (121). For example, a previous study found *Ube3a*^*m*−*/p*+^ mice did not make any USVs in a 5-minute courtship test (116). We extended testing to 20 min and confirmed that while we saw a phenotype in both the first 10 min and the second 10 min of the test, most USVs indeed occurred in the test’s second half **(Additional File 2: Supplementary Fig. 2)**. Performing a 20-minute test provides a richer dataset by giving the male mice more time to interact with the female and respond and is more similar to the length of courtship vocalization testing in other studies that can range from 10 min to 5 hours (59, 74, 96, 122–124). Second, a potential confound in testing USVs using a three-chamber behavioral arena is that if the WT female spends more time interacting with the WT male, the lack of proximity may account for why *Ube3a*^*m*−*/p*+^ males make fewer calls. We predicted that WT females would spend less time with *Ube3a*^*m*−*/p*+^ males, but in fact females spent similar amounts of time in the WT and *Ube3a*^*m*−*/p*+^ chambers **(Additional File 3: Supplementary Fig. 3).** Female preference may take longer than 20 min, so if this is an important behavioral outcome, lengthening the test is recommended. However, in a 20-minute test, exposure time to the female is unlikely to explain the decreased calls in *Ube3a*^*m*−*/p*+^ males. Third, we also stress the importance of habituating mice to reduce anxiety and novel object exploration, such as biting and climbing on the plexiglass cages. Finally, other courtship considerations include using littermate males to minimize scent differences that could affect female preference and secondary analysis of audio files to identify calls picked up by both microphones **(Additional File 1: Supplementary Fig. 1)**. The distress test requires no habituation, but all of our tests were performed handling naïve mice, so handling exposure may be an important consideration. Overall, while methodological considerations are important, our results and prior work demonstrate that impaired USVs are a consistent feature of *Ube3a*^*m*−*/p*+^ mice in a wide range of behavioral contexts.

## CONCLUSIONS

Adult *Ube3a*^*m*−*/p*+^ mice show a clear vocalization deficit in both social courtship and nonsocial distress contexts. Vocal features of courtship calls are more impacted than vocal features of distress calls, but distress testing is easy and robust. Adding the 60-second distress test to the existing Angelman behavioral battery would be beneficial for testing preclinical therapeutics.

## Data Availability

The datasets used and/or analyzed during the current study are available from the corresponding author upon reasonable request.

## Abbreviations

AS: Angelman syndrome
PCA: Principal component analysis
PUMBAA: Phenotyping Using a Multidimensional Behavioral Analysis Algorithm
SV: Syllable vocalization
USV: Ultrasonic vocalization
WT: Wild-type
